# Environmental Silica Dust Exposure and Pulmonary Tuberculosis in Johannesburg, South Africa

**DOI:** 10.3390/ijerph16101867

**Published:** 2019-05-27

**Authors:** Tahira Kootbodien, Samantha Iyaloo, Kerry Wilson, Nisha Naicker, Spo Kgalamono, Tanya Haman, Angela Mathee, David Rees

**Affiliations:** 1 National Institute for Occupational Health, National Health Laboratory Service, Constitution Hill, Johannesburg 2001, South Africa; SamanthaI@nioh.ac.za (S.I.); KerryW@nioh.ac.za (K.W.); NishaN@nioh.ac.za (N.N.); SpoK@nioh.ac.za (S.K.); DavidR@nioh.ac.za (D.R.); 2 School of Public Health, Faculty of Health Sciences, University of the Witwatersrand, Parktown 2193, South Africa; amathee@mrc.ac.za; 3 Environmental Health Department, Faculty of Health Sciences, University of Johannesburg, Johannesburg 2000, South Africa; taneeh@gmail.com; 4 Environment and Health Research Unit, South African Medical Research Council, Johannesburg 2000, South Africa

**Keywords:** pulmonary tuberculosis, occupational dust exposure, silica, silica-related tuberculosis, occupational history

## Abstract

Background: Occupational crystalline silica dust exposure is associated with an elevated risk of pulmonary tuberculosis (PTB). However, there is less evidence for an association with environmental silica dust exposure. Methods: A cross-sectional study of 310 participants was conducted in an exposed community living within 2 km of gold mine tailings and an unexposed population residing more than 10 km from the nearest gold mine tailing. Chest radiographs (*n* = 178) were read for PTB, past or current, by three readers. Results: Past or current PTB was radiologically identified in 14.4% (95%CI 9.2–21.8) in the exposed and 7.5% (95%CI 2.8–18.7) in the unexposed groups. Multivariate logistic regression models suggested that PTB prevalence was independently associated with exposure to second-hand smoke (OR = 8.13, 95%CI 1.16–57.22), a lower body mass index (OR = 0.88, 95%CI 0.80–0.98), previous diagnosis and treatment of PTB (OR = 8.98, 95%CI 1.98–40.34), and exposure to dust in the workplace from sand, construction, and/or mining industries (OR = 10.2, 95%CI 2.10–50.11). Conclusion: We found no association between PTB and environmental exposure to gold mine tailing dust. However, workplace silica dust exposure is a significant risk factor for PTB in South Africa, and PTB patients of working age should be screened for silica exposure.

## 1. Introduction

Past gold mining activities have generated substantial amounts of waste material in South Africa. Nearly 300 gold mine tailings situated near highly urbanised areas or close to valuable agricultural land have been identified [1]. Environmental studies on mining operations and the reclamation and erosion of existing mine tailings in the Witwatersrand area of Johannesburg have reported contamination of adjacent land with metals, salts and radionuclides [2,3]. Additionally, high levels of crystalline silica (silica from now on) have been found in the Witwatersrand gold mine tailings and surrounding areas [4]. Concern about the potential impact of dust from gold mine tailings on the health of neighbouring communities in South Africa has been growing [5,6]. 

Long-term silica dust exposure is associated with an increased risk of respiratory diseases such as silicosis, lung cancer, chronic obstructive pulmonary disease, and pulmonary tuberculosis (PTB) [7]. Pulmonary tuberculosis (PTB) is potentially the predominant public health issue in silica-exposed South African communities because, despite a 9% decline in tuberculosis (TB) incidence from 2008 to 2012, TB remains the leading infectious cause of death in the country [8]. 

The association between silica exposure and TB is well established in occupational settings [9,10,11], but less is known about environmental exposures and associated health effects in communities living close to silica-generating workplaces or mine tailings. In India, elevated mean ambient PM_10_ silica concentrations were reported in areas near a pencil slate (41.1 µg/m^3^) and agate industry (57.2 µg/m^3^) compared to the control site 5 km away (3.5 µg/m^3^) [12]. More recently, in Gauteng Province, South Africa, Andraos et al. reported elevated ambient PM_10_ crystalline silica levels in communities surrounding gold mine tailings [4]. 

TB is a significant public health burden that causes considerable morbidity and mortality in South Africa. This study is to the best of our knowledge the first investigating the association between PTB and environmental sources of silica-rich dust in communities living near gold mine tailings. The study aimed to determine the prevalence of past or current PTB and the associations with potential environmental silica dust exposure and other risk factors. 

## 2. Materials and Methods

### 2.1. Study Population and Setting

**Figure 1:**
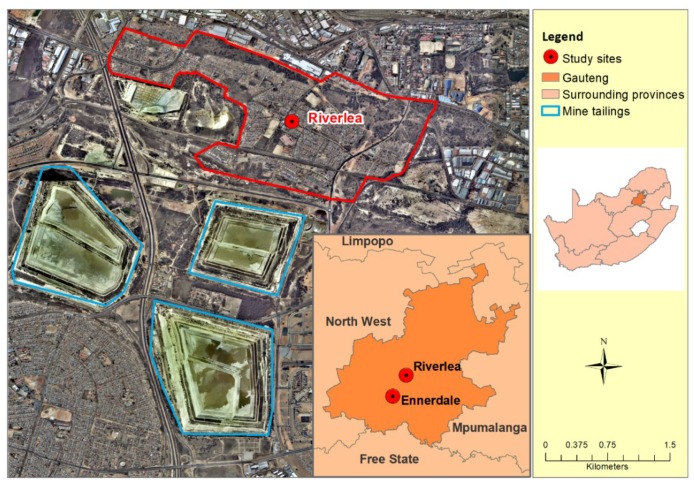
Map of the study area showing the study sites and proximity to gold mine tailings.

This cross-sectional household study was conducted in two urban communities in Johannesburg, South Africa and is part of a larger household survey investigating the association between dust exposure and respiratory health. The current study reports on TB findings only. The two study sites were formal housing communities with similar socioeconomic status (based on the percentage of people living in poverty) [13]. Three large gold mine tailings surround the exposed community (Riverlea) with the nearest tailing 200 m away (Figure 1). The three mine tailings close to Riverlea are partially covered with vegetation. However, due to dry conditions, successful rehabilitation of gold mine tailings remains challenging. Based on weather station data (from 1994 to 2016) representing wind data at Riverlea from the South African Weather Service, the average annual prevailing wind direction was easterly, with a tendency for flows to concentrate in north-west and north-easterly directions [4], suggesting that the wind could carry dust from the tailings towards Riverlea. The unexposed population (Ennerdale) is more than 10 km from the nearest gold mine tailing.

**Figure 2:**
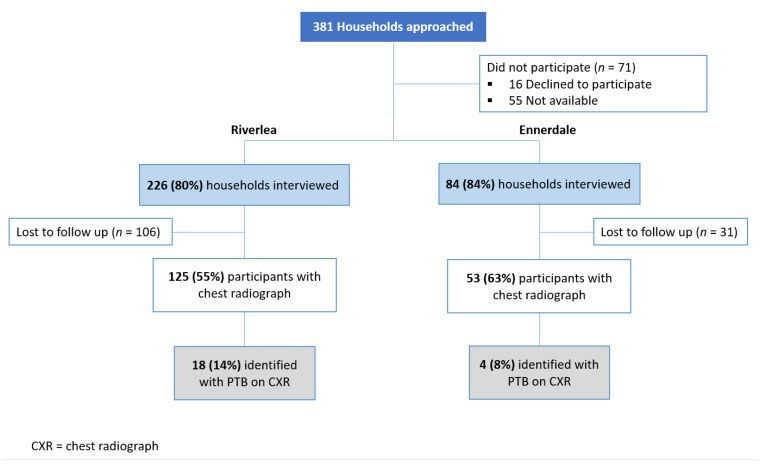
Flow chart describing study recruitment and enrolment.

We selected a random sample of households in the study area using Erf or stand numbers from cadastral maps purchased from the City of Johannesburg. An Erf or stand number is a number allocated to identify each land parcel in the City. Of the 381 households approached, 310 participants, aged 18 years and older, completed interviews. All participants were invited to return for a chest radiograph two weeks later; 178 (57.4%) did so, as shown in Figure 2. Informed written consent was obtained before completing the interview and conducting chest radiographs. A clinical assessment of PTB was done by professional nurses, and if TB was suspected, participants were immediately referred to the local clinic. All 22 participants with radiological diagnosis of PTB were contacted and referred to their local clinic for bacteriological diagnosis and treatment. This study was approved by the Human Research Ethics Committee (Medical), University of the Witwatersrand (Reference number M150550).

### 2.2. Measures

Household and individual information were collected using a structured questionnaire on a handheld electronic device. Interviewers were trained in interviewing techniques and field work processes. Exposure to gold mine tailing dust was categorised according to study site (Riverlea = yes; Ennerdale = no). Data were collected on socio-demographic information (age, sex, education level, socio-economic status, employment status, overcrowding, and monthly income), smoking history, exposure to secondary smoke, occupational history (working in a dusty job, duration of employment and type of dust exposure), environmental dust exposure (years living in the study site, perception of outdoor dust in windy conditions and indoor dust), history of chronic illnesses, and body mass index (BMI). 

### 2.3. Radiological Assessment of Tuberculosis

Our primary interest was past or current PTB. The usefulness of chest radiographs for the screening of PTB especially among asymptomatic TB cases has been demonstrated in several recent prevalence studies [14]. Full-sized posterio-anterior radiographs were taken by the South African National Accreditation System (SANAS) approved service providers, who were trained radiographers. Radiographs were independently assessed by a radiologist and two experienced readers at the National Institute of Occupational Health (NIOH). The readers classified TB as present or absent. Where only two of the three readers agreed, we used majority opinion (median read) to classify TB cases. Silicosis was defined as rounded opacities with a profusion of 1/0 or more reported by at least two of the three readers according to the International Labour Organization’s Classification of Radiographs of Pneumoconioses [15].

### 2.4. Statistical Analysis

Data were analysed using STATA 14 (Stata Corp., College Station, TX, USA). The primary outcome variable was a PTB diagnosis on chest radiograph. BMI was calculated using weight/(height)^2^. The number of people in the household was divided by the number of bedrooms to determine whether conditions in the household were crowded. Overcrowding was defined as more than three people per bedroom. Univariate and multivariate logistic regression analyses were used to test for factors (socio-demographic characteristics, occupational history, and self-reported medical history) associated with PTB. As conditions of poverty are both a cause and a consequence of PTB, we considered the following factors in the model: BMI (reviewed by Lönnroth et al. [16]); low or no income and overcrowding (reviewed by Duarte et al. [17]); tobacco smoke exposure and use of biomass fuel (reviewed by Lin et al. [18]). Effect modification between community proximity to mine tailings and other sources of air pollution was also investigated, i.e. the use of biomass fuel for cooking and heating, occupational silica dust exposure and exposure to second-hand smoke. Types of occupational dust exposure and work in a dusty job were collinear. We used types of occupational dust exposure in the model building as it provided more information on dust exposure. Additional variables were included in the model if p-values were < 0.1 in the univariate analysis. Goodness-of-fit of the final model was assessed using the Hosmer–Lemeshow test. The kappa statistic and 95% confidence intervals were calculated to evaluate inter-reader agreement for PTB identified on chest radiographs and between the readers and a positive history of PTB. 

## 3. Results

Table 1 provides the socio-demographic characteristics, occupational and medical history of 178 participants, aged between 19 to 81 years, who were screened for PTB. The median age in the total sample was 51 years. In Riverlea (exposed group), the study participants were older (53 years vs. 45 years); had a longer-term residence in the area (32 years vs. 17 years) and had a higher proportion of overcrowding (14% vs. 1%) compared to participants living in Ennerdale (unexposed group). A substantial proportion of Riverlea participants worked in a dusty environment for more than a year (29% vs. 19%) and were exposed to dust from construction and mining industries (10% vs. 4%). 

Approximately 93% of participants reported having a kitchen as a separate room. Participants from Ennerdale reported using more biomass fuel for cooking and heating (25% vs. 9%). There were no differences by smoking history or pack-years smoked between the groups. However, a larger proportion of the Riverlea participants who were non-smokers reported exposure to second-hand smoke (8% vs. 4%). The medical history was similar for the two groups except that a higher percentage of Ennerdale subjects reported a previous diagnosis of PTB (11.5% vs. 6.6%). Overcrowded households were associated with higher average monthly income (nptrend, *p* = 0.002) and weakly correlated with a higher BMI (Spearman rho 0.15, *p* = 0.04).

Radiologic signs consistent with past or current PTB were identified in 22 participants (12.4%, 95% CI 8.2–18.1) with prevalences of 14.4% (95% CI 9.2–21.8) in Riverlea and 8.0% (95% CI 2.8–18.7) in Ennerdale (Figure 2). Kappa statistics revealed a very good agreement between the three readers for the detection of PTB on chest radiograph (kappa 0.82, 95% CI 0.70–0.91) [19]. No cases of silicosis were found. 

Factors potentially associated with PTB in 178 participants who had a chest radiograph are summarised in Table 2. In the univariate analysis, elevated PTB prevalence was associated with older age (Odds ratio, OR = 1.03, 95% CI 1.00–1.07), exposure to second hand smoke (OR = 4.11, 95% CI 1.12–15.07), working in a dusty environment for more than a year (OR = 2.88, 95% CI 1.14–7.22), exposure to dust in the workplace from sand, construction and/or mining (OR = 5.51, 95% CI 1.58–19.11), a previous history of PTB (OR = 7.76, 95% CI 2.36–25.59) and a low body mass index (OR = 0.92, 95% CI 0.85–0.99).

In the multivariate analysis (Table 3), PTB prevalence was associated with exposure to second-hand smoke (OR = 8.13, 95% CI 1.16–57.22), a lower body mass index (OR = 0.88, 95% CI 0.80–0.98), previous diagnosis and treatment of PTB (OR = 8.98, 95% CI 1.98–40.34) and exposure to dust in the workplace from sand, construction and/or mining industries (OR = 10.2, 95% CI 2.10–50.11).

## 4. Discussion

The health effects of environmental exposure to silica-rich dust in communities living near gold mine tailings is a public health concern in South Africa [4]. In our analysis, we found no statistically significant association between PTB prevalence and living near gold mine tailings. This is despite reports of other dust-related health effects in these communities [4,5,20].

We found a high prevalence of radiological PTB (12.2%). At present, there are no nationally representative TB prevalences available, but the 12.2% is similar to the radiologically-detected PTB prevalence (12.9%) in two low socioeconomic communities in Cape Town, Western Cape Province, which had the highest TB notification rate in South Africa [21]. We found an association between PTB and second-hand smoke among non-smokers. Patra et al. [22] reviewed 12 studies and reported an almost 2-fold increased risk of active PTB from second-hand exposure and an increased risk of latent TB infection (pooled RR 1.67, 95% CI 1.12–2.48). This finding was confirmed in a review by Bai et al. (2018) who reported that the susceptibility for latent and active TB among those exposed to second-hand smoke may be singly or in combination due to suppressed anti-TB immunity and enhanced activity of immunosuppressive N2 neutrophils [23]. Second-hand smoke is a growing concern in regions where PTB poses a significant health risk. Despite the stringent tobacco control policies in place in South Africa, more is needed to ensure that communities are protected from second-hand smoke and the subsequent risk of PTB. 

PTB is more prevalent among older populations. Therefore, older populations are more likely to have reported past and/or current PTB diagnosis. Thus, the older population found in Riverlea is considered a strength of the study. Older TB patients are more susceptible to infection due to changes in the immune system related to ageing [24], indicating the need for a high index of suspicion in elderly patients. 

We found no association between PTB and the use of biomass fuel. This may have been due to the selection of community controls who reported a higher prevalence of biomass fuel use. Also, the majority of participants reported having a separate kitchen to cook in and may have had adequate ventilation. Future studies should include measures of indoor air pollution and exposure duration. Several socio-economic factors such as overcrowding, poverty, and income inequality are associated with PTB. However, we did not report an association between overcrowding and PTB. In our study, crowded households contained adults of working age and were associated with higher average household monthly income and a higher BMI. Thus, crowded households were less likely to be at increased risk of PTB. A further limitation included a small sample size.

In this study, occupational exposure to dust was significantly associated with the prevalence of PTB, mainly dust from the construction and mining industries. The relationship between occupational silica exposure, even in the absence of radiologic silicosis, and TB has been firmly established [9,10]. Silica-related TB can be prevented provided that airborne exposures are minimised, and workers are protected against inhalation [25]. Nevertheless, historical overexposure and current poor dust control in many workplaces mean that cases of occupational silica-induced TB will occur for decades to come. 

These findings show that occupational history is therefore critical at primary health care level in everyday clinical practice. A detailed occupational history is necessary to identify at-risk groups when screening for TB. Workplace acquired TB is an important occupational disease among workers exposed to silica, such as construction [26] and gold mine workers [10]. Additionally, workers with silica-induced TB are eligible for workers’ compensation in South Africa [27]; TB patients of working age should be questioned about silica exposure and submitted for compensation as appropriate.

### Study Limitations

Some limitations should be acknowledged when interpreting the study findings. 

The study had limited power to show a statistically significant association between environmental exposure to mine tailings dust and PTB because of the relatively small sample size. We cannot conclude, therefore, that such an association does not exist, especially since an OR of 2.06 was found. A larger study might have resulted in a significant association. 

TB cases were diagnosed by radiological assessment rather than microbiological evidence. Under-reading and over-reading of PTB on chest radiographs are well documented [28]. Despite the limitation that chest radiographs are not TB-specific, it is considered a sensitive tool for the screening of active PTB [29] and is recommended for detecting PTB in prevalence surveys [30]. Despite the overall good agreement among the chest radiograph readers, this may have resulted in over or under-diagnosis of TB disease. However, the findings of associations between established risk factors and PTB do provide reassurance that if misclassification of disease exists in the study, it was not large enough to obscure strong positive associations. 

We did not measure environmental silica dust levels. However, Andraos et al. (2018) reported elevated PM_10_ and PM_4_ silica levels and showed an increased risk of non-cancerous health effects in communities surrounding gold mine tailings near our study area [4]. Air pollution from outdoor sources such as industrial activities may have contributed to the increased TB prevalence in Riverlea. Based on the close proximity of Riverlea to industrial sources of air pollution, further research is needed to disentangle the association between outdoor air pollution and TB. We did not offer HIV testing in our study but relied on self-reporting. The very small proportion of participants who provided information on HIV positive status could have been because of the stigma associated with HIV in our setting. 

The effect of the relatively low response rate for chest radiograph of 57.4% is difficult to interpret. Possibly subjects with symptoms related to past or current PTB would be more likely to present for chest radiograph than those without the disease. This would falsely elevate the prevalence of PTB in our study population. It seems unlikely that PTB symptoms would differentially affect Riverlea and Ennerdale, though, so this potential bias would not explain the lack of a significant association between PTB and exposure to tailings dust (living in Riverlea). 

## 5. Conclusions

Environmental exposure to silica-containing dust was not associated with TB in this study, but a larger study with more refined exposure data is needed to confirm this finding. We found an association with PTB and reported silica exposure in the workplace. Besides primary prevention through dust control, obtaining an occupational history at primary health care level when screening for TB should be emphasised. Special populations, such as workers exposed to silica dust, should be among those prioritised for active case finding in high burden communities.

## Figures and Tables

**Table 1:** Description of the study population (*n* = 178).

Characteristics	Riverlea * n (%)	Ennerdale * n (%)	*p*-Value
*N*	125	53	
**Socio-demographic**			
Median age in years (IQR)	53 (23–79)	45 (20–66)	0.006
Female %	64.8	62.3	0.346
Average monthly income			
No income	22 (17.9)	9 (16.9)	
<R 1000	15 (12.2)	3 (5.7)	
≥R 1000	86 (69.9)	41 (77.4)	0.397
Overcrowding (>3 persons per bedroom)	18 (14.4)	1 (1.9)	0.013
Biomass fuel used for cooking/heating	11 (8.8)	13 (24.5)	0.009
Smoking history	70 (56.5)	29 (54.7)	0.831
Total pack-years smoked			
Non-smoker (<0.5)	52 (41.6)	23 (43.4)	
0.5–10	32 (25.6)	19 (35.8)	
11–20	21 (16.8)	7 (13.2)	
>21	20 (16.0)	4 (7.6)	0.299
Exposed to second-hand smoke	10 (8.0)	2 (3.8)	0.304
**Environmental dust exposure**			
Median years living in the study area (IQR)	32 (8–60)	17 (5–39)	<0.001
Perception of outdoor dust during windy weather	114 (92.7)	48 (90.6)	0.635
Dust inside the house	81 (65.9)	36 (67.9)	0.789
**Occupational dust exposure**			
Worked in a dusty environment > 1 year	35 (28.5)	10 (18.9)	0.181
Median years worked in a dusty environment (IQR)	6.0 (1–37)	5.5 (5–10)	0.365
Types of dust exposure			
No dust exposure	88 (71.5)	43 (81.1)	
Wood/saw dust	13 (10.6)	2 (3.8)	
Metal/welding	6 (4.9)	1 (1.9)	
Sand/construction/mining	12 (9.8)	2 (3.8)	
Other	4 (3.2)	5 (9.4)	0.102
**Medical history**			
Median BMI (kg/m^2^)	26.3 (16.6–44.7)	27.2 (18.3–39.0)	0.609
Previous diagnosis of PTB	8 (6.6)	6 (11.5)	0.276
Self-reported diagnosis of diabetes (*n*, %)	12 (9.6)	4 (7.6)	0.661
Self-reported HIV status (*n*, %)	2 (1.6)	2 (3.8)	0.371

* Riverlea = exposed to gold mine tailings dust; Ennerdale = not exposed.

**Table 2:** Univariate analysis of socio-demographic, occupational and clinical factors associated with radiological pulmonary tuberculosis (PTB).

Characteristic	*n*	Crude OR (95% CI)	*p*-Value
**Socio-demographic**			
Age in years	178	1.03 (1.00–1.07)	0.033
Sex			
Female	64	Reference	
Male	114	1.94 (0.79–4.78)	0.147
Average monthly income			
≥R 1000	127	Reference	
No income	31	3.19 (1.17–8.66)	0.051
<R 1000	18	1.90 (0.48–7.58)	0.354
Overcrowding (≥3 persons/ bedroom)			
No	159	Reference	
Yes	19	1.38 (0.37–5.19)	0.632
Biomass fuel used for cooking/ heating			
No	154	Reference	
Yes	24	1.51 (0.52–5.97)	0.493
Smoking history			
Non-smoker	78	Reference	
Smoker	99	1.78 (0.57–3.63)	0.216
Total pack-years smoked			
Non-smoker (<0.5)	75	Reference	
0.5–10	51	1.25 (0.42–3.75)	0.285
11–19	28	1.61 (0.43–6.02)	0.472
≥20	24	1.38 (0.33–5.84)	0.655
Exposed to second-hand smoke			
No	166	Reference	
Yes	12	4.11 (1.12–15.07)	0.033
BMI (kg/m^2^)	178	0.92 (0.85–0.99)	0.030
**Environmental dust exposure**			
Exposure to gold mine tailing dust			
No (Ennerdale)	53	Reference	
Yes (Riverlea)	125	2.06 (0.66–6.41)	0.212
Years living in the study area	178	0.69 (0.25–1.91)	0.480
Dust inside the home			
No	59	Reference	
Yes	117	1.09 (0.42–2.85)	0.859
**Occupational dust exposure**			
Worked in a dusty environment >1 year			
No	133	Reference	
Yes	45	2.88 (1.14–7.22)	0.024
Types of dust exposure			
No dust exposure	131	Reference	
Wood/saw dust	15	1.52 (0.30–7.57)	0.605
Metal/welding	7	1.65 (0.18–14.89)	0.654
Sand/construction/mining	14	5.51 (1.58–19.11)	0.007
Other	9	2.83 (0.52–15.20)	0.224
Years worked in a dusty environment	178	1.02 (0.98–1.07)	0.307
**Medical history**			
Self-reported HIV status			
Negative	174	Reference	
Positive	4	7.38 (0.98–55.20)	0.052
Self-reported diagnosis of diabetes			
No	161	Reference	
Yes	16	0.41 (0.05–3.28)	0.404
Previous diagnosis of TB			
No	163	Reference	
Yes	14	7.76 (2.36–25.59)	0.001

**Table 3:** Multivariate logistic regression analysis of factors associated with radiological PTB.

Characteristic	*n*	Adjusted OR * (95% CI)	*p*-Value
**Socio-demographic**			
Exposure to gold mine tailing dust			
No (Ennerdale)	53	Reference	
Yes (Riverlea)	125	2.02 (0.35–11.48)	0.423
Mean age in years (SD)	178	1.04 (0.99–1.09)	0.106
Average monthly income			
≥R 1000	127	Reference	
No income	31	3.19 (0.85–11.97)	0.085
<R 1000	18	0.81 (0.48–7.58)	0.826
Exposed to second-hand smoke			
No	166	Reference	
Yes	12	8.13 (1.16–57.22)	0.035
BMI (kg/m^2^)	178	0.88 (0.80–0.98)	0.017
**Occupational history**			
Types of dust exposure			
No dust exposure	131	Reference	
Wood/saw dust	15	0.78 (0.11–5.56)	0.808
Metal/welding	7	0.82 (0.05–12.35)	0.884
Sand/construction/mining	14	10.2 (2.10–50.11)	0.004
Other	9	7.42 (0.83–65.7)	0.071
**Medical history**			
Previous diagnosis of TB			
No	163		
Yes	14	8.98 (1.98–40.34)	0.004

* Model fit: Hosmer-Lemeshow X^2^ (8) = 13.96, *p* = 0.283.
